# Analysis of *Fusobacterium nucleatum* Driven Modulation of c-Myc Pathways in Oral Carcinogenesis

**DOI:** 10.12688/f1000research.177011.1

**Published:** 2026-02-03

**Authors:** Charu Priya, Anshi Jain, Devi Charan Shetty, Atrey J. Pai Khot, Shefali Yadav, Mahima Jain, Nikita Gulati, Sultan A. Almalki, Inderjit M. Gowdar

**Affiliations:** 1 Department of Oral and Maxillofacial Pathology and Microbiology, ITS Centre for Dental Studies and Research, Ghaziabad, Uttar Pradesh, India; 2 Department of Public Health Dentistry, Goa Dental College and Hospital, Bambolim, Goa, India; 3 Department of Preventive Dental Sciences, College of Dentistry, Prince Sattam bin Abdulaziz University, Alkharj, Saudi Arabia

**Keywords:** Fusobacterium nucleatum, c-Myc expression, Oral squamous cell carcinoma (OSCC), Oral potentially malignant disorders (OPMDs), Oral microbiome, Carcinogenesis

## Abstract

**Objective:**

To evaluate the association between *Fusobacterium nucleatum* and c-Myc expression in Oral Potentially Malignant Disorders (OPMDs) and Oral Squamous Cell Carcinoma (OSCC) and to explore its potential role in oral carcinogenesis.

**Materials and Methods:**

A total of 32 histopathologically confirmed cases (18 OPMDs and 14 OSCC) were analyzed. Anaerobic cultures and polymerase chain reaction (PCR) were used to detect *F. nucleatum.* Immunohistochemistry (IHC) was performed to assess c-Myc expression. Statistical analysis was conducted using Mann-Whitney and Chi-square tests, with p < 0.05 considered significant.

**Results:**

*F. nucleatum* was detected in eight OSCC and two OPMD cases, with higher colony counts in OSCC. All samples were positive for c-Myc, but their expression levels varied. In OPMDs, positivity was mainly observed in the basal and suprabasal epithelial layers, whereas OSCC showed both peripheral and central tumor cell localization. *F. nucleatum*–positive OSCC cases demonstrated strong nuclear c-Myc staining (50–75% positive cells). Tobacco habits, particularly combined smoking and smokeless use, were more common in *F. nucleatum*–positive OSCC cases.

**Conclusion:**

*F. nucleatum* colonization correlates with increased c-Myc expression in OPMDs and OSCC, supporting its possible role in microbially driven oral carcinogenesis. These findings suggest its potential as a prognostic biomarker and a therapeutic target.

## Introduction

Head and neck cancer is a complex disease encompassing variable pathology, genetics, and tissue biology that can be subjected to multifaceted treatment response. ^1^ Five percent of all malignancies are head and neck cancers, where oral squamous cell carcinoma (OSCC) stands out. Despite recent advancements in treatment, identification and prevention it continues to pose a serious threat worldwide. ^2^ The multifactorial process of oral carcinogenesis includes the impact of the exposome and subsequent cytogenetic and epigenetic alterations in keratinocytes. ^3^ These effects could be brought about by certain risk factors such as tobacco in all forms, alcohol, u-v radiation, viruses and evolution from certain oral potentially malignant disorders (OPMDs). Oral leukoplakia, erythroplakia, oral submucous fibrosis (OSMF), and proliferative verrucous leukoplakia are a few examples of lesions with dysplastic traits that have been labelled as OPMDs. ^4^

In 2022, Hanahan added polymorphic microbiomes as one of the emerging hallmarks and enabling characteristics. ^1^ The components of oral microbiota including bacteria, fungi, and viruses, as emerging hallmarks and enabling characteristics. A stable microbial community exists in a healthy environment; however, under certain conditions, microbial homeostasis can be upset, culminating in the emergence of dysbiosis, which is characterized by an increase in the proportion of bacteria with the capacity to cause disease or an increase in the production of virulence factors. They are known to stimulate chronic inflammation, which facilitates cell proliferation, mutagenesis, oncogene activation, and angiogenesis by activating anti-apoptotic activity and forming reactive oxygen and nitrogen species. ^5^

*Fusobacterium nucleatum, * a subspecies of *Fusobacterium* is a gram-negative, anaerobic, non-sporing, non-motile, pleomorphic, filamentous bacillus that commonly inhabits the human oral cavity. It is known to form dental plaque and is considered a pivotal bridging bacterium for the attachment of commensals that colonize the tooth and the epithelial surface. ^6, 7^ They replicate effectively in the hypoxic tumor microenvironment, and their colonization begins early in the process of malignant transformation. *F. nucleatum* stimulates tumorigenesis by directly acting on epithelial cells through their Toll-like receptors (TLR), which results in the production of IL-6, which activates STAT3, which in turn induces cyclin D1, c-Myc, matrixmatalloproteases-9 (MMP-9), which are important effectors for the growth and invasiveness of OSCC cells. They also stimulate cell proliferation by upregulating c-Myc via STAT3 and interacting with endothelial cadherin(E-cadherin) through FadA, which in turn activates WNT/β-catenin signalling, resulting in cell proliferation through increased expression of c-Myc (Figure S1). ^5, 8^

MYC genes are a family of proto-oncogenes located on chromosomes 8q21and and comprised several members, such as L-Myc, C-Myc, and N-Myc, which are indispensable in cell proliferation regulation, differentiation, and apoptosis. These are found in normal cells and encode proteins in the cell nucleus that bind to DNA, facilitating transcription and regulating the activity of other cells involved in cell division. ^9, 10^ c-Myc gene has been found to be overexpressed in OSCC associated with significantly poor prognosis and self-renewal of stem cells. ^9^ Keeping this in mind, the present study aimed to explore the *Fusobacterium nucleatum* mediated c-Myc pathway in the progression of OSCC.

## Materials and methods

### Study design and sample collection

This prospective study was conducted with ethical approval from the institutional board of the Department of Oral and Maxillofacial Pathology and Microbiology, I.T.S Dental College, Muradnagar, Ghaziabad (Ref. No: ITSCDSR/IIEC/LD/2021-24/OP/01). A total 32 samples were included in the study, comprising 18 cases of OPMDs and 14 cases of OSCC. OSCC cases were further subgrouped into well-differentiated Oral Squamous Cell Carcinoma (WDSCC) and moderately differentiated Oral Squamous Cell Carcinoma (MDSCC). A written informed consent was obtained from all the subjects involved in the study. Demographic details, including age, sex, site of lesion, and habit history (type, duration, and frequency) for each case, were obtained. Tissue samples from each patient were obtained for immunohistochemical analysis, swab samples from the same patients were taken for microbial culture, and further genomic analysis from microbial colony growth was performed. Swab samples were stored immediately in sterile sample collection vials containing phosphate buffer saline (chilled 1X PBS) solution at -20 ^ °^C deep freezer for further processing. Histopathologically confirmed cases of OSCC and OPMDs were processed for microbial cultures.

The inclusion criteria were clinically confirmed cases of OSCC and OPMDs, Patients whose clinical details were available. exclusion criteria cases with any therapeutic intervention were excluded. Immunocompromised patients with debilitating diseases. Patients who were not ready to undergo biopsy.

### Bacterial culture and DNA extraction

Swabs were vortexed, serially diluted, and inoculated onto Lombard–Dowell agar plates. The plates were incubated anaerobically (N _2_ 85%, H _2_ 5%, CO _2_ 10%) at 37 °C for 4–5 days. The colony-forming units (CFUs) were counted and expressed as CFU/mL. Colonies suspected to be Fusobacterium nucleatum were harvested for DNA extraction using a bacterial genomic DNA purification kit. PCR was performed using species-specific primers (forward, 5′-GGTTCAGAAGTAGGACCGGGAGA-3′; reverse, 5′-ACTCCCTTAGAGCCATGAGGCAT-3′). *F. nucleatum* ATCC 25586 was used as the positive control.

### Immunohistochemistry

The 4 μm sections were obtained from formalin-fixed paraffin-embedded specimens on polylysine-coated slides. Immunohistochemical staining was performed using the streptavidin-biotin-peroxidase complex method. The slides were then incubated with the primary antibody c-Myc (mouse monoclonal antibody, Biogenex AM318-5M) at room temperature for 1 h. Diaminobenzidine was used as the chromogen. Normal oral mucosa was used as a positive control. All immunostained slides were viewed under a light microscope at high power (40x). Positive staining for c-Myc indicated crisp brown nuclear and/or cytoplasmic localization. Immunoscoring for positive cells for c-Myc was done score 1- <25% cells, score 2-25-50% cells, score 3-50-75% cells, score 4- >75% cells. ^11^ For qualitative analysis tissue section was observed under the light microscope at 100X and 400X for intensity of staining and scored as weak positive, moderate positive, strong weak. ^11^

### Statistical analysis

Data were analyzed using IBM Corp. 2012, IBM Corp., Armonk, NY, Version 21.0. Armonk, NY: IBM Corp. Descriptive statistics were calculated. Group differences in CFU counts and PCR results were assessed using the chi-square test, while correlations were tested using Pearson’s chi-square test. Statistical significance was set at P < 0.05.

## Results

**Figure 1.**
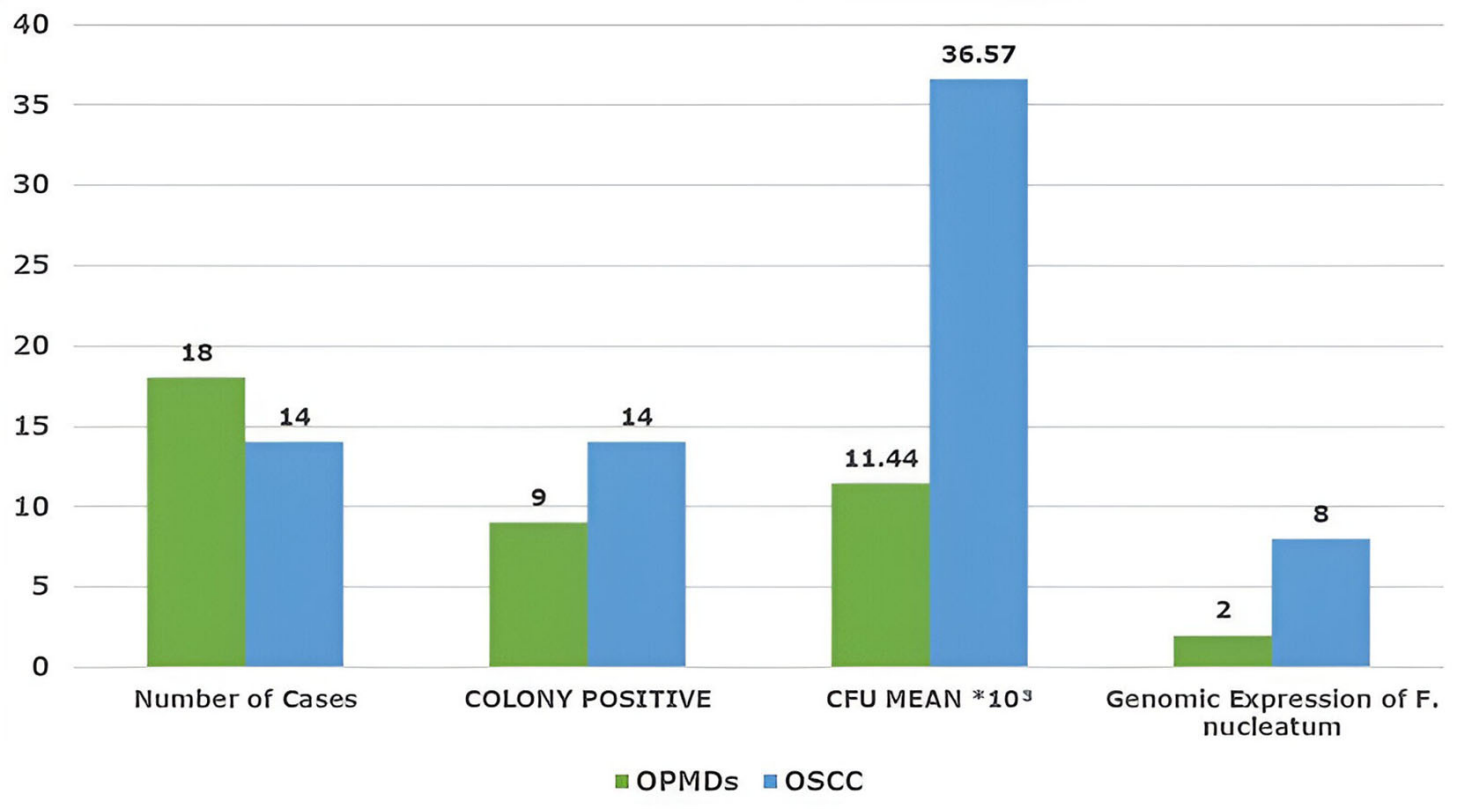
Microbiologically and genomically positive cases of F. Nucleatum in OPMDs and OSCC groups.

**Figure 2.**
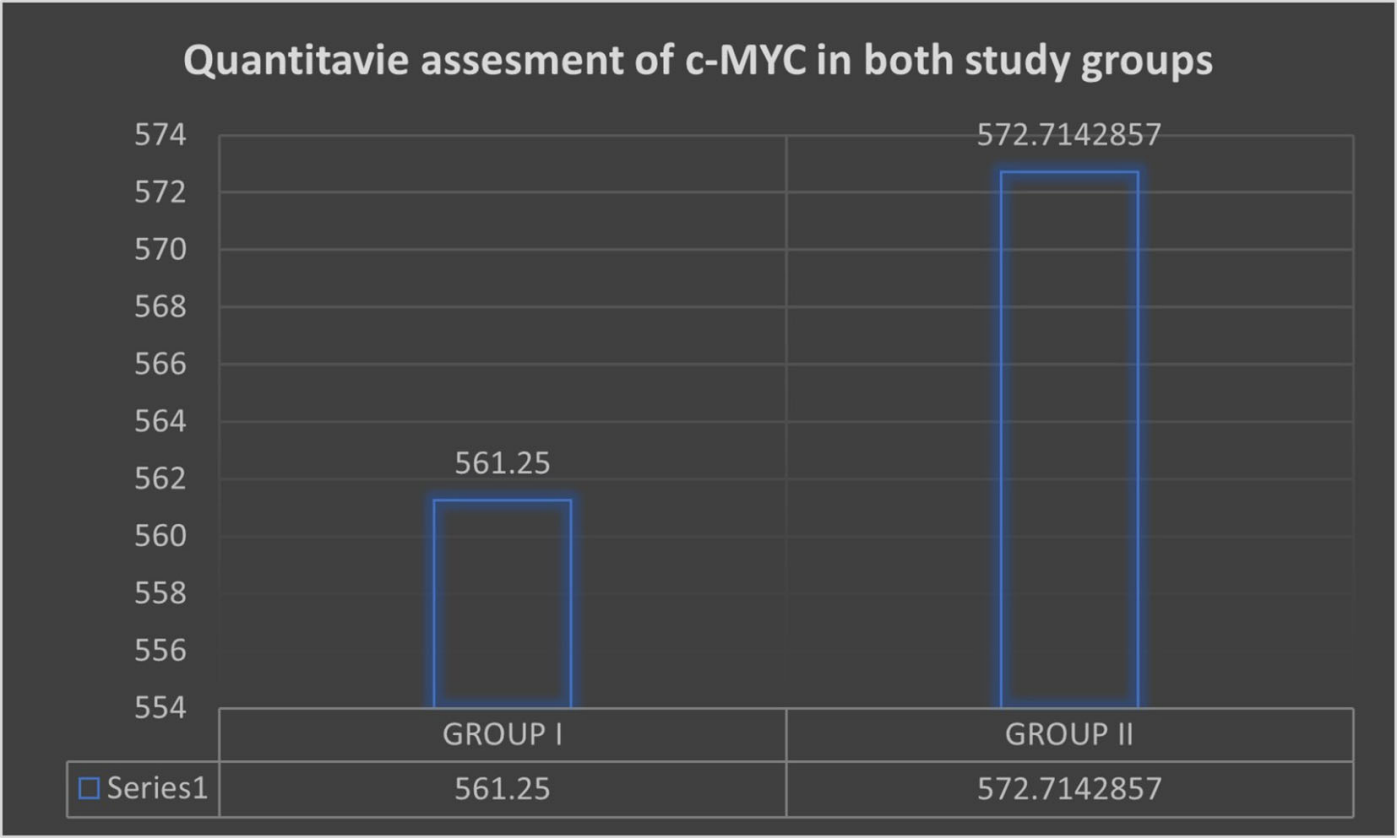
Quantitative assessment of both the study groups.

**Figure 3.**
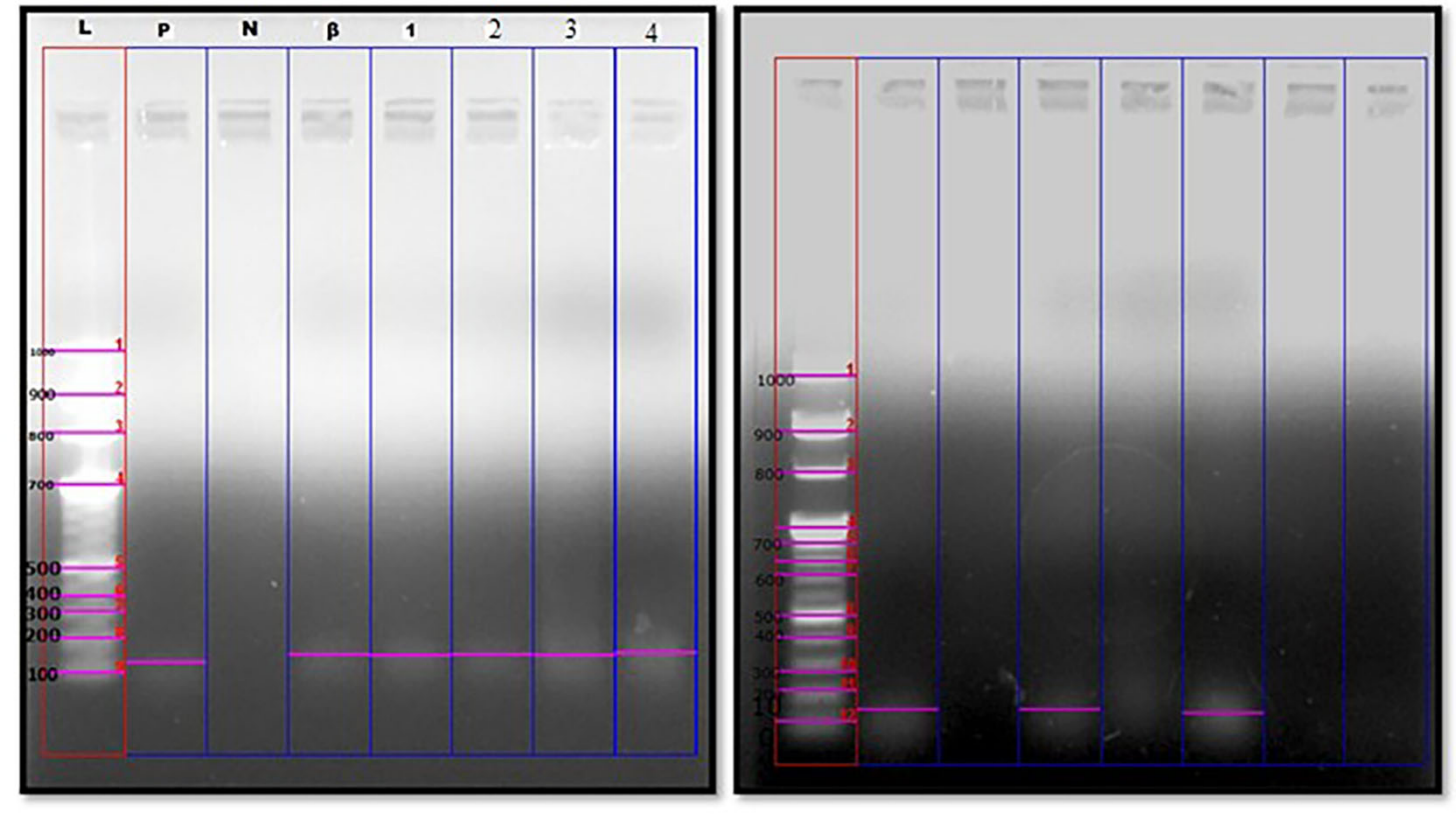
Genomic amplification of F. nucleatum in OSCC and OPMDs cases at 161 bp. L-DNA ladder at 100bp; P-Positive control; N-Negative control; β-beta globin housekeeping gene; 1-4: Samples. A. Positive genomic expression of Fusobacterium nucleatum in OSCC samples at 161 bp. B. Positive genomic expression of Fusobacterium nucleatum in OPMD’s samples at 161 bp.

**Figure 4.**
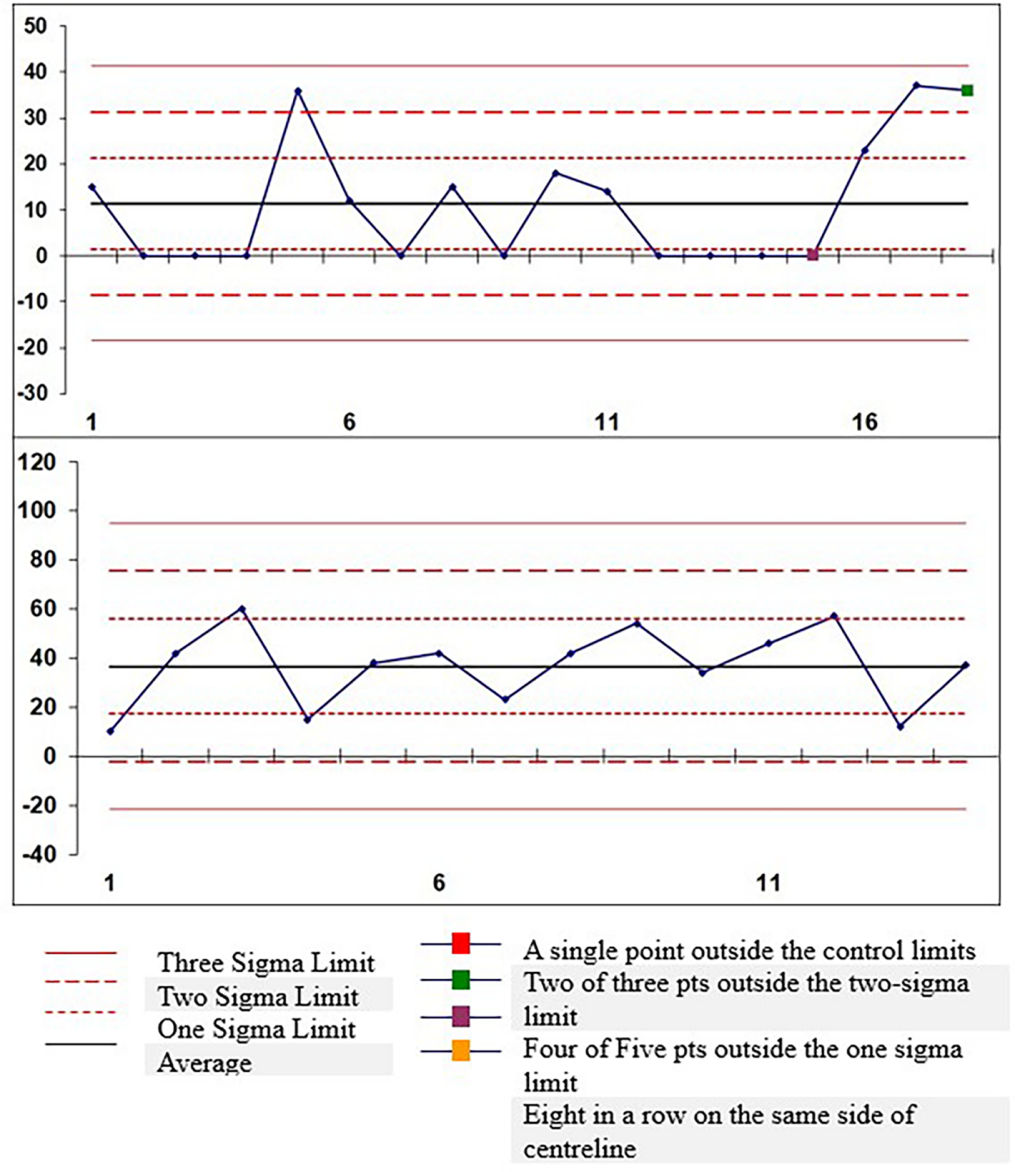
The graph depicts the colony growth in 32 cases of Group -I (OPMDs) and Group- II (OSCC).

**Figure 5.**
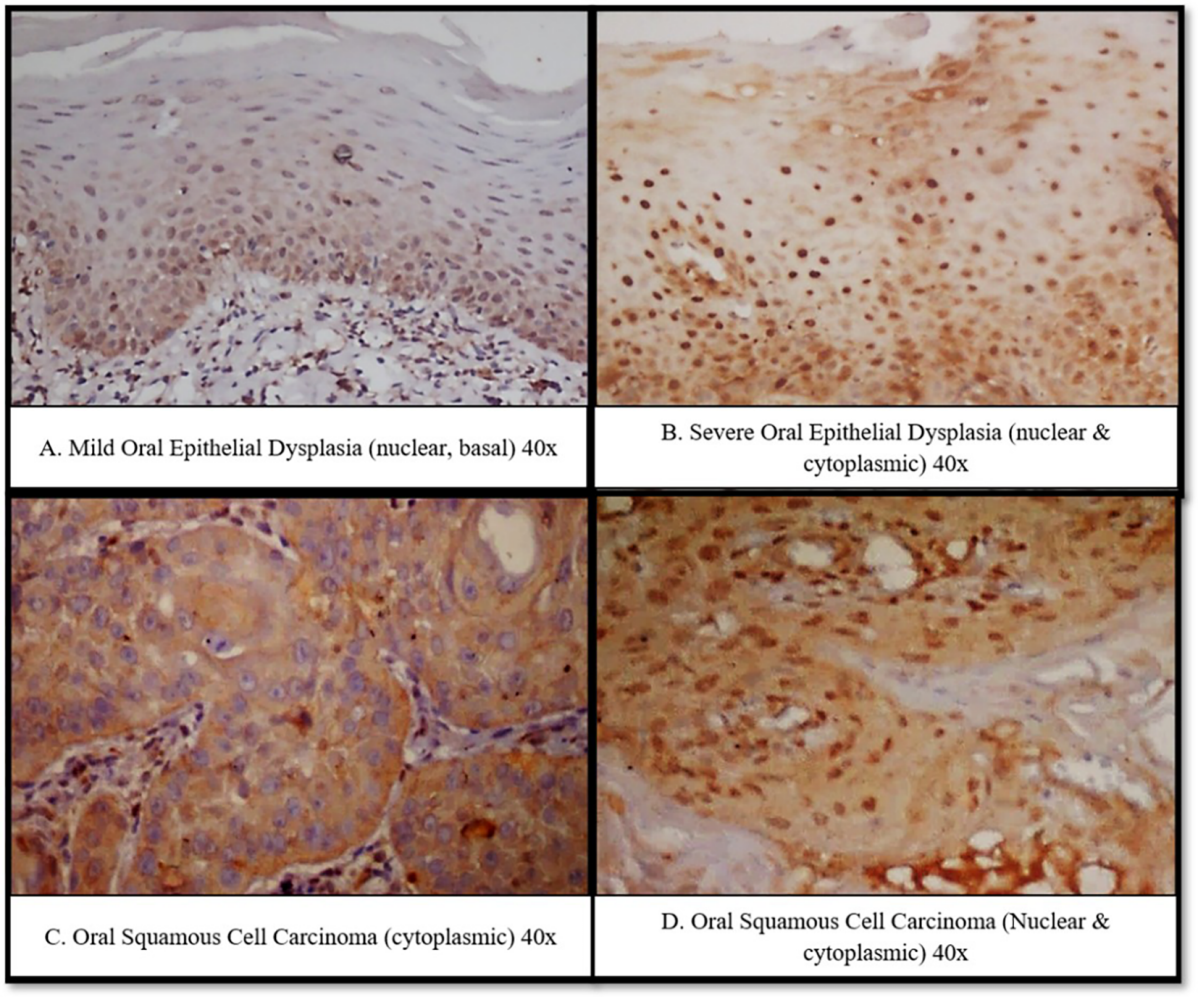
Immunoexpression of c-Myc in A. Mild Oral Epithelial Dysplasia (nuclear, basal) 40x. B. Severe Oral Epithelial Dysplasia (nuclear & cytoplasmic) 40x. C. Oral Squamous Cell Carcinoma (cytoplasmic) 40x. D. Oral Squamous Cell Carcinoma (Nuclear & cytoplasmic) 40x.

A total of 32 cases were evaluated, including 18 OPMDs (Group I) and 14 OSCC (Group II). Clinical and demographic parameters, such as age, sex, habit type, quantity, frequency, and duration, showed no statistically significant differences between the groups (  Table 1). Most participants in both groups were male and above 30 years of age. Site distribution differed significantly between the groups (p = 0.00). OPMDs were predominantly located in the left buccal mucosa (55.5%), while OSCC cases most commonly involved the mandibular posterior region (42.8%) and retromolar trigone (35.7%). Immunohistochemical evaluation demonstrated increased c-Myc expression in OSCC, with a higher proportion of strongly positive cases (42.8%) than that in OPMDs (11.1%) (  Table 2). Semi-quantitative analysis showed a statistically significant difference (p = 0.009), with OSCC showing higher expression levels (50–75% staining in 78.5% of the cases). Genomic analysis revealed Fusobacterium nucleatum positivity in both groups, although it was more prominent in OSCC. Immunolocalization patterns differed significantly (p = 0.026), with OSCC showing increased cytoplasmic and combined nuclear–cytoplasmic c-Myc localization. Microbiological culture confirmed anaerobic colony growth in 23 of 32 cases, aligning with PCR-based positivity for F. nucleatum. Habit correlation among F. nucleatum–positive cases suggested higher positivity in individuals with smokeless tobacco use, higher consumption (>2 bundles/day), and longer habit duration (>15 years), predominantly in OSCC cases (  Table 3). As shown in  Figure 1, all cases of OSCC and a few cases of OPMDs showed anaerobic growth with mean colony count of 36.57*103 and 11.44*103, respectively. The mean quantitative score for c-MYC expression is shown in Figure 2. c-MYC expression was highest in Group II (572.71), followed by Group I (561.25 in OSMF cases. Furthermore, on genomic analysis, only two cases of OPMDs and eight cases of OSCC were positive for F. nucleatum (  Figure 3). Out of eight cases of OSCC, five and three cases were WDSCC and MDSCC, respectively. Of the two cases of OPMD, one case was of Severe Oral epithelial dysplasia (SOED) (mean CFU 19.25*103) and the other was of oral submucosal fibrosis (OSMF) (mean CFU 14 × 103). The maximum number of F. nucleatum-positive cases of OPMDs (n = 2) and OSCC (n = 5) showed moderate immunoexpression of c-Myc. Case-wise control analysis of anaerobic microbial colony count showed better results in the OSCC study group. Of the 32 cases, 23 were positive for colony growth (  Figure 4). Fn-positive cases demonstrated predominant nuclear c-Myc staining, either peripherally or throughout the tumor islands (  Figure 5).

**Table 1.** Correlating immunohistochemical expression of c-myc and genomic analysis of F. Nucleatum in OPMDS and OSCC.

CHARACTERSTICS		STUDY GROUPS (n = 32)		Pearson chi-square	p-value
CRITERIA	CATEGORY	GROUP-I (OPMDs n = 18)	GROUP-II (OSCC n = 14)		
**AGE**	**<30 YEARS**	5(27.7%)	1(7.14%)	2.201	0.138 NS
	**>30 YEARS**	13(72.2%)	13(92.8%)		
**GENDER**	**MALE**	16(88.8%)	13(92.8%)	0.146	0.702 NS
	**FEMALE**	2(11.1%)	1(7.14%)		
**SITE**	**RIGHT BUCCAL MUCOSA**	8(44.4%)	-	32	0 NS
	**LEFT BUCCAL MUCOSA**	10(55.5%)	-		
	**MAXILLARY LEFT PALATAL REGION**	-	2(14.2%)		
	**MANDIBULAR POSTERIOR**	-	6(42.8%)		
	**LEFT LATERAL BORDER OF THE TONGUE**	-	1(7.14%)		
	**RETROMOLAR TRIGONE**	-	5(35.7%)		
**HABIT TYPE**	**TOBACCO SMOKLESS**	9(50.0%)	5(50.0%)	4.717	0.194 NS
	**TOBACCO SMOKING**	4(22.2%)	2(14.2%)		
	**BOTH**	1(5.55%)	5(35.7%)		
	**NO HABIT**	4(22.2%)	2(14.2%)		
**HABIT QUANTITY**	**<2 BUNDLES/PACKET**	6(33.3%)	5(35.7%)	1.669	0.434 NS
	**>2 BUNDLES/PACKET**	8(44.4%)	7(50.0%)		
	**NONE**	4(22.2%)	2(14.2%)		
**HABIT FREQUENCY**	**<5 TIMES/DAY**	10(55.5%)	7(50.0%)	0.82	0.664 NS
	**>5 TIMES/DAY**	4(22.2%)	5(35.7%)		
	**NONE**	4(22.2%)	2(14.2%)		
**HABIT DURATION (YEARS)**	**<15 YEARS**	9(50.0%)	7(50.0%)	0.423	0.809 NS
	**>15 YEARS**	5(27.7%)	5(35.7%)		
	**NONE**	4(22.2%)	2(14.2%)		

All values are expressed as frequencies and percentages (parentheses). Statistical tests were performed using the chi-squared test.

NS - non-significant.

**Table 2.** Correlating immunohistochemical expression of C-Myc and genomic analysis of F. Nucleatum in OPMDS And OSCC.

CHARACTERSTICS		STUDY GROUPS (n = 32)				p-value
CRITERIA	CATEGORY	GROUP-I (OPMDs n = 18)	F. nucleatum positive	GROUP-II (OSCC n = 14)	F. nucleatum positive	
**QUALITATIVE**	**WEAK POSITIVE**	4(22.2%)		1(7.14%)		0.245
	**MODERATE POSITIVE**	12(66.6%)	2	8(57.1%)	5	
	**STRONGLY POSITIVE**	2(11.1%)		6(42.8%)	3	
**SEMI-QUANTITATIVE **	**<25%**	-		-		**0.009** ** **HS**
	**25-50%**	10(55.5%)		1(7.14%)		
	**50-75%**	5(27.7%)	1	11(78.5%)	6	
	**>75%**	3(16.6%)	1	2(14.2%)	2	
**TOPOGRAPHIC**	**BASAL**	5(27.7%)		-		0
	**BASAL+SUPRABASAL**	5(27.7%)		-		
	**BASAL+SUPRA+SUPERFICIAL**	8(44.4%)	2	-		
	**PERIPHERAL**	-		4(28.5%)	4	
	**CENTRAL**	-		-	4	
	**PERIPHERAL+CENTRAL**	-		10(71.4%)		
**IMMUNO-LOCALIZATION **	**CYTOPLASMIC**	-		4(28.5%)	3	0.026 * S
	**NUCLEUS**	16(88.8%)	1	7(50.0%)	4	
	**BOTH**	2(11.1%)	1	3(21.4%)	1	

All values are expressed as frequencies and percentages (parentheses). Statistical tests were performed using the chi-squared test. Level of significance:

* p ≤ 0.05, considered statistically significant;

** p ≤ 0.001, highly significant association; HS -Highly significant; S- Significant.

**Table 3.** Habit correlation of F. Nucleatum positive cases in the study groups.

GROUPS		PCR for F. nucleatum	HABIT	QUANTITY	FREQUENCY	DURATION
**OPMDs**	** OSMF**	Positive	Smoking	<2 bundles	>5 times/day	<15 years
	**SEVERE OED**	Positive	Both	<2 bundles	<5 times/day	<15 years
**OSCC**	**WDOSCC**	Positive	Both	>2 bundles	<5 times/day	>15 years
	**MDOSCC**	Positive	Smokeless	>2 bundles	>5 times/day	>15 years
	**WDOSCC**	Positive	Both	>2 bundles	<5 times/day	<15 years
	**WDOSCC**	Positive	Smokeless	>2 bundles	>5 times/day	<15 years
	**WDOSCC**	Positive	Both	<2 bundles	>5 times/day	>15 years
	**MDOSCC**	Positive	Smokeless	>2 bundles	<5 times/day	<15 years
	**MDOSCC**	Positive	Both	>2 bundles	<5 times/day	>15 years
	**WDOSCC**	Positive	Both	>2 bundles	<5 times/day	<15 years

PCR, Polymerase Chain Reaction; F. nucleatum, Fusobacterium nucleatum; OPMDs, Oral potentially malignant disorders; OSMF, Oral submucosal fibrosis; OED, Oral epithelial dysplasia; OSCC, Oral squamous cell carcinoma; WDOSCC, Well-differentiated oral squamous cell carcinoma.

## Discussion

The oral cavity contains more than 700 microbial species that collectively maintain mucosal equilibrium but may also contribute to carcinogenesis when dysbiosis occurs. Recent studies have emphasized that oral commensals can drive oral squamous cell carcinoma (OSCC) through genetic and epigenetic mechanisms. ^12, 13^

Al-Hebshi et al in 2019 introduced the “passenger-to-driver” model, proposing that a dysbiotic intratumoral microbiota sustains cancer development. ^14^ Among the implicated species, *Fusobacterium nucleatum* ( *Fn*) has emerged as a potent “driver,” interacting directly with epithelial cells to promote tumorigenesis. ^8^ According to Hanahan, 2022 ^1^ Hallmarks of Cancer: New Dimensions -Polymorphic Microbiome has been additionally proposed as the emerging hallmark and enabling characteristics. This hallmark of cancer influences its other hallmarks. There is growing appreciation that ecosystems created by resident bacteria and fungi (microbiomes) have a profound impact on health and disease. Current diverse translations with respect to population exposure have resulted in inevitable multifold disease ramifications of bacterial infections.

Therefore, the present study was conducted to emphasize the role of F. nucleatum in the progression of Oral Squamous Cell Carcinoma by cellular proliferation through upregulation of c-Myc. This study included 32 cases (18 cases of OPMDs and 14 cases of OSCC). In both study groups, the majority of cases reported were >30 years of age and predominantly male (  Table 1). This was in accordance with the study conducted by Borse et al. (2020). ^15^

According to Singh AK et al. (2021) ^16^ most common site for OPMDs are buccal mucosa and vestibule with habit of chewing tobacco which was in accordance with our study Group -I (OPMDs) with 18(100%) cases seen at site of buccal mucosa with (50.0%) cases with habit of chewing tobacco. In Group II (OSCC), the common site was the buccal mucosa (42.8%), with a habit of both smoking and smokeless tobacco 35.7% this finding was in accordance with the findings of Singh et al. (2016) ^17^ and Tandon et al. (2018). ^18^

In a study conducted by Sakai et al. (1990), ^19^ 100% of the tumors expressed c-Myc oncoprotein. Our results are in accordance with those of the present study, as in our study groups c-Myc immunoexpression was positive in all 32 cases (100%) (  Table 2). The c-Myc staining pattern was classified as cytoplasmic, nuclear, or both. The present study showed variable localization patterns, among which nuclear immunolocalization was dominant in both the study groups (88.8%-OPMDs, 50.0%-OSCC) (Graph 2), whereas only 28.5% of OSCC cases revealed cytoplasmic immunolocalization followed by (11.1%) OPMDs, and (21.4%) of OSCC cases revealed both cytoplasmic and nuclear immunolocalization. Ectopic expression of c-Myc is sufficient for cell cycle progression. Nuclear positivity was dominant in the majority of cases that showed larger nuclei and pleomorphism (  Figure 2). Segura et al. (2013) ^20^ reported similar findings for nuclear overexpression in 73% of OSCC cases. There are no studies in the literature that differentiate between the cytoplasmic and nuclear localization of c-Myc in OPMDs. Pérez-Sayáns et al. (2011) ^21^ and Segura et al. (2013) ^20^ studied nuclear and cytoplasmic localization and reported differential localization of c-Myc in OSCC.

Our results illustrate significant variation in the immunoexpression of c-Myc among various layers of the dysplastic epithelium of OPMDs and different compartments of the tumor islands (OSCC) (  Table 2). The majority of cases (44.4%) among OPMDs showed c-Myc immunoexpression in the basal, suprabasal, and superficial layers, and the expression proportionately increased among the increasing grades of dysplasia. Among OSCCs (71.4%), c-Myc immunoexpression was predominantly seen in both the peripheral and central cell layers. In dysplasia, c-Myc positivity proportionally involved all strata of the epithelium in accordance with the grade. Baral et al. (1998), ^22^ Papakosta et al. (2009), ^23^ Bhattacharya et al. (2006), ^24^ and Pallavi et al. (2018) ^25^ are in agreement with the results of the present study. Eversole and Sapp (1993) ^26^ reported parallel results for precancerous and cancerous lesions. They found that in cases of dysplasia, carcinoma in situ, and carcinoma, c-Myc nuclear expression was dominant in all strata harboring atypical cells, and the degree of staining intensity increased as the level of atypia increased. These results indicated that the c-Myc oncoprotein is expressed in the basal layer of oral leukoplakia that exhibits hyperkeratosis without cytological atypia. This could be due to the secretion of mitogenic or cytokine factors from subepithelial leukocytes, which are produced as an inflammatory response towards epithelial dysplasia. They also revealed that the labelling of the spinous nuclei was increased in severe dysplasia, indicating that once the cells exhibit top-to-bottom atypia, they have reached their full malignant potential. Altered and high variability in the expression of c-Myc in different layers is a probable indicator for exploring transcriptional activities and their instability in causation.

According to Dang et al. (1999) ^27^ the cytoplasmic positivity of c-Myc is because c-Myc needs to dimerize with Max to bind to E-box to activate the downstream gene in transformed cells, and accumulation of c-Myc protein in the cytoplasm might suggest an unknown deregulation pathway in promoting tumor growth. Therefore, cytoplasmic immunolocalization of c-Myc expression indicates a worse prognosis than a typical nuclear pattern.

*Fn* colonization was markedly higher in OSCC than in OPMDs, consistent with earlier reports of Nagy et al in 1998, Al-Hebshi et al in 2017, Zhao et al in 2017. ^28– 30^ Approximately 22% of OSCC cases showed *Fn* positivity (Graph 1), similar to Zhang L et al in 2020, who found Fusobacteriaceae dominance (>25%) in cancerous lesions. ^31^ Severe oral epithelial dysplasia (19.25 × 10 ^3^ CFU) and oral submucous fibrosis (14 × 10 ^3^ CFU) revealed the highest *Fn* counts, confirming previous findings that *Fn* enrichment accompanies epithelial dysplasia. ^32^

*Fn*-positive cases demonstrated predominant nuclear c-Myc staining, either peripherally or throughout the tumor islands (  Figure 2). Such nuclear localization corresponds to increased cellular atypia, whereas cytoplasmic accumulation may signal disrupted Max dimerization or abnormal downstream activation. ^33^ Cytoplasmic expression is often associated with poorly differentiated tumors and poor prognosis. ^34^

In the present study, we correlated habit with positive cases of Fusobacterium nucleatum (  Table 3). OSMF and Severe OED have a habit of smoking; the latter have a habit of taking both smokeless and smoking tobacco. These results were in accordance with the results of the study by Wu et al. (2016) ^35^ stated that smoking is related to an overall increase in oral microbiome community composition. They commented that smoking causes an anaerobic environment that alters oral microbial ecology by influencing oxygen availability by decreasing the oxygen content in the oral mucosa. They also commented that there is depletion in certain xenobiotic biodegradation pathways, as oral bacteria are first to come into contact with cigarette smoke as they enter the human body and may play an important role in degrading the accompanying toxic compounds; thus, alterations in the ability of the oral community to degrade these substances may have toxic consequences for the host. They also commented that some xenobiotic degradation pathways are depleted in smokers, given the need for bacterial upregulation of these pathways to detoxify cigarette smoke.

In the study by Zhang et al. (2018) ^36^ suggested that there are three mechanisms of action of oral microbiota in the pathogenesis of cancer: 1) stimulation of chronic inflammation, which causes or facilitates cell proliferation, mutagenesis, oncogene activation, and angiogenesis. 2) It affects cell proliferation, cytoskeletal rearrangements, activation of NF-κB, and inhibition of cellular apoptosis (anti-apoptotic activity). 3) by producing carcinogenic substances such as reactive oxygen species, reactive nitrogen species, and reactive volatile sulfur compounds ( Figure 3).

There are 13 papers in the literature on the association between oral premalignant disorders, mainly leukoplakia, and oral microbiota. According to the review of these papers done by Pietrobon G et al. (2021) ^37^ stated two theories “bacteria before tumour” and “bacteria after tumour.” Before the tumor, bacteria damage the epithelial cells, which activate a cascade of inflammatory pathways, leading to cell replication and reactive oxygen species (ROS) production, ultimately leading to DNA damage and carcinogenesis. Bacteria after tumors suggest that opportunistic bacteria are attracted by the hypoxic, hyper-vascularized tumor environment, and they sustain the progression of the unhealthy ecosystem, leading to the further evolution of carcinogenesis.

Taken together, our findings support a model in which *Fn* colonization progressively increases from OPMDs to OSCC, driving c-Myc overexpression and altering its localization pattern in association with disease progression. This microbial–molecular interplay may represent an important early event in malignant transformation. Clinically, these results underscore the potential of *Fn* as a biomarker for risk stratification in OPMDs and as a therapeutic target in OSCC. Strategies aimed at modulating oral dysbiosis or inhibiting downstream oncogenic signalling, including the c-Myc pathway, could provide novel avenues for prevention and treatment.

However, the limitations of our study must be acknowledged. The sample size was modest, particularly for the OPMD group, in where only two cases tested positive for *Fn.* This limits the generalizability of our results and warrants further validation in larger cohorts. Additionally, although c-Myc positivity is universal, its expression is not specific to *Fn* and may be influenced by multiple alternative pathways. Further studies employing multi-marker panels, functional assays, and metagenomic profiling are needed to confirm the direct causal role of *Fn* in c-Myc-mediated carcinogenesis.

## Conclusion

Our results support that Fusobacterium nucleatum acts not only as a passive colonizer but also as a biological modulator enhancing malignant potential through c-Myc-mediated pathways. F. nucleatum colonization correlates with increased c-Myc expression in OPMDs and OSCC, supporting its possible role in microbially driven oral carcinogenesis. These findings suggest its potential as a prognostic biomarker and a therapeutic target. Routine assessment of the microbial profiles in OPMDs could serve as an adjunctive diagnostic tool for identifying lesions at a higher risk of transformation. Targeting Fn-related inflammatory and signalling pathways may open new avenues for microbiome-based preventive or therapeutic interventions for OSCC.

## Ethics approval

This study was conducted in accordance with the Declaration of Helsinki and approved by the Internal Institutional Ethical Committee I.T.S Centre for Dental Studies and Research under the protocol number ITSCDSR/IIEC/LD/2021-24/OP/01.

## Informed consent statement

A written informed consent was obtained from all the subjects involved in the study.

## Data Availability

All underlying de-identified data generated and analyzed in this study are openly available in Figshare, an F1000Research-approved generalist data repository. The dataset includes all relevant cleaned data files and documentation necessary for verification and reuse. Data were shared under the terms of the Creative Commons Attribution 4.0 International (CC-BY 4.0) license, permitting unrestricted use, distribution, and reproduction provided by the original authors. The dataset can be accessed at the following DOI: 10.6084/m9.figshare.30885599 ^38^ The Supplementary Figure S1 can be accessed at the following DOI -10.6084/m9.figshare.31082239 ^39^
