# Supplementary material for: Analysis of Fusobacterium nucleatum Driven Modulation of c-Myc Pathways in Oral Carcinogenesis
Source: F1000Res. 2026 Feb 3;15:176. [Version 1] doi: 10.12688/f1000research.177011.1 (PMC12959620; doi:10.12688/f1000research.177011.1)
Supplement: Peer review history

# Reviewer report: Reviewer response for version 1

**DOI:** 10.5256/f1000research.195151.r455451

**Published:** 2026-03-02

**Authors:** Sufi Mary Thomas, Molly Muehlebach

**Affiliations:** 1 The University of Kansas Medical Center, Kansas City, Kansas, USA

This article aims to elucidate a novel mechanism by which the oral microbiome (specifically F. nucleatum) may contribute to oral disease pathogenesis and provides a clear contribution to the field of oral cancer. The data provided characterize the presence of Fusobacterium nucleatum in oral tissue from 32 patients (18 cases of oral premalignant disease (OPMD), and 14 cases of oral squamous cell carcinoma (OSCC)). In addition, the expression and intracellular localization of c-Myc was characterized in all cases. The study describes well the expression and localization of c-Myc and prevalence of Fn in the samples. All the cases expressed c-Myc, while 22% of OSCC were Fn positive. In Fn positive OSCC, c-Myc was predominantly in the nucleus. The authors also examined the association of tobacco use and Fn incidence.

 Immunohistochemical staining data have not been included making it difficult to evaluate the localization of c-Myc in Fn positive cases. The conclusions are overstated relative to the data presented. There is no evidence to suggest that “Fn drives c-Myc overexpression, altering its localization pattern in association with disease progression”. It is possible that Fn prevalence and c-Myc nuclear localization may be true but unrelated. In addition, the conclusion that “F. nucleatum colonization correlates with increased c-Myc expression” is unsupported by the data presented. A 100% of the cases express c-Myc while only 22% of OSCC are positive for Fn. In addition, because no normal oral mucosa control is included, claims of c‑Myc overexpression cannot be substantiated. As such, even the title needs to be edited to reflect the data as presented. Overall, the manuscript needs major revisions.

 Please see recommendations for edits below:

  

1. Introduction, paragraph 2, sentence 1 – add “of cancer” to the end of the sentence for clarification. Following sentence is repetitive of sentence 1, recommend rephrasing.
2. Introduction, paragraph 4, sentence 1 – recommended edit to “and comprised of several members” instead of “comprised several members…”
3. Introduction, paragraph 4, sentence 3 – recommend citing primary research articles were OSCC overexpression is correlated to poor prognosis and stem cell renewal rather than citing a review article.
4. Materials and methods, subsection “Study design and sample collection”, paragraph 2 – Description of inclusion and exclusion criteria is unclear. Please fix sentence structure.
5. Materials and methods, subsection “Immunohistochemistry” – I believe there is a typo in the final sentence of this section. Please change “strong weak” to “strong positive” if correct.
6. Results, Table 1 – recommend changing the description of this table as the data does not represent immunohistochemical scoring of c-myc expression.
7. Results, Table 2 – error in table 2. Please move F. nucleatum positive (4) in the topographic section to the peripheral+central row if correct.
8. Results, paragraph 1 – In the results section, the authors state “Habit correlation among F. nucleatum–positive cases suggested higher positivity in individuals with smokeless tobacco use, higher consumption (>2 bundles/day), and longer habit duration (>15 years), predominantly in OSCC cases”. This is not definitively clear in the data shown in table 3 since all reported cases regardless of tobacco use (both <2 bundles/day and >2 bundles/day) were F. nucleatum positive. Furthermore, the F. nucleatum status of non-tobacco users was not included. I think it would be helpful to show this data if you want to make the point that F. nucleatum prevalence is related to tobacco use.
9. Results, Figure 1- is difficult to interpret as presented. There is no Y axis label.
10. Results, Figure 2 –for clarity, maintain consistent labels for group 1 and 2 as mentioned Tables 1 and 2. Include a Y axis label. How was the quantitative assessment carried out? The scoring seems to be qualitative. The authors need to generate a composite score for each sample by multiplying the percent positivity with the intensity of stain for each sample. Was statistics done on this data? If so, please state whether staining was statistically different between groups. Provide representative images of the two groups.
11. Results, Figure 3. Is confusing as presented. The pink line over the data needs to be removed as it interferes with assessment. An arrow of the side of the gel with the molecular weight to indicate the expected band would suffice. There are two gels but the label for only the first gel is clarified in the legend. There are no A and B labels so it is not clear what the legend is referring to. The bands are very blurred lacking definition. It looks like primer diners. The quality of the gel is poor. To demonstrate equal loading of samples across all lanes the housekeeping gene needs to be assessed across all samples. Clarify what the nature of the positive and negative controls in the legend. It is not clear how the data were generated to ensure that there is no bacterial DNA contamination in the samples.
12. Results, Figure 4 – Figure is difficult to interpret as presented. It is not clear what the authors mean by colony growth. Is it the number of colonies, rate of growth, or the size? Add a label to the x and y-axis to describe what the data points are representative of. Label group 1 and 2 on the graph. Further explanation is needed for the coloured squares mentioned in the legend.
13. Results, Figure 5 – provide IHC data from normal oral mucosa. Include data from Fn positive and negative cases. Include data to substantiate “Fn drives c-Myc overexpression, altering its localization pattern in association with disease progression”.

Is the work clearly and accurately presented and does it cite the current literature?

Partly

If applicable, is the statistical analysis and its interpretation appropriate?

Yes

Are all the source data underlying the results available to ensure full reproducibility?

No source data required

Is the study design appropriate and is the work technically sound?

Yes

Are the conclusions drawn adequately supported by the results?

Partly

Are sufficient details of methods and analysis provided to allow replication by others?

Yes

Reviewer Expertise:

Head and neck squamous cell carcinoma, oral microbiome in cancer pathogenesis

We confirm that we have read this submission and believe that we have an appropriate level of expertise to confirm that it is of an acceptable scientific standard, however we have significant reservations, as outlined above.

# Reviewer report: Reviewer response for version 1

**DOI:** 10.5256/f1000research.195151.r455456

**Published:** 2026-02-26

**Authors:** Deepak Gowda Sadashivappa Pateel

**Affiliations:** 1 Mahsa University, Selangor, Malaysia

This manuscript examines the correlation between Fusobacterium nucleatum colonization and c-Myc expression in oral potentially malignant disorders and oral squamous cell carcinoma, addressing a pertinent biological issue, as the role of the microbiome in cancer becomes increasingly acknowledged, and aiming to connect microbial dysbiosis with oncogenic molecular mechanisms in a conceptually robust manner.

 The manuscript is well-structured and logically organized, with clear objectives and relevant background literature.

 The introduction provides a comprehensive overview of the oral microbiome's role in cancer progression.

 - A brief clarification regarding the IHC scoring methodology would enhance reproducibility.

 -The conclusion is stronger for the correlation study. change accordingly in the text

 - Briefly highlight future research directions in the conclusion.

 - Mann–Whitney mentioned in the abstract but not clearly presented in the results.

Is the work clearly and accurately presented and does it cite the current literature?

Yes

If applicable, is the statistical analysis and its interpretation appropriate?

Yes

Are all the source data underlying the results available to ensure full reproducibility?

Yes

Is the study design appropriate and is the work technically sound?

Yes

Are the conclusions drawn adequately supported by the results?

Yes

Are sufficient details of methods and analysis provided to allow replication by others?

Yes

Reviewer Expertise:

ORAL PATHOLOGY, ORAL MEDICINE, ORAL BIOLOGY, ORAL CANCER, SALIVARY GLAND RESEARCH, DENTAL EDUCATION RESEARCH.

I confirm that I have read this submission and believe that I have an appropriate level of expertise to confirm that it is of an acceptable scientific standard.

# Reviewer report: Reviewer response for version 1

**DOI:** 10.5256/f1000research.195151.r455452

**Published:** 2026-02-23

**Authors:** Durgesh Bangalore

**Affiliations:** 1 King Saud University, Riyadh, Riyadh Province, Saudi Arabia

Overall Assessment

 The manuscript is well-presented and addresses a clinically significant topic. The authors provide detailed methodology and clear statistical descriptions, making the study easy to follow.

Strengths

 •    Tables summarizing clinicopathological correlations are informative and clearly organized.

 •    The manuscript demonstrates strong clinical relevance, particularly regarding potential prognostic biomarkers.

 •    The conclusions are aligned with the presented data and emphasize translational implications.

Minor Suggestions

 •    Minor grammatical corrections may further enhance clarity.

 •    Ensure uniform reporting of statistical values (e.g., consistent decimal formatting for p-values).

Is the work clearly and accurately presented and does it cite the current literature?

Yes

If applicable, is the statistical analysis and its interpretation appropriate?

Yes

Are all the source data underlying the results available to ensure full reproducibility?

Yes

Is the study design appropriate and is the work technically sound?

Yes

Are the conclusions drawn adequately supported by the results?

Yes

Are sufficient details of methods and analysis provided to allow replication by others?

Yes

Reviewer Expertise:

Dentistry

I confirm that I have read this submission and believe that I have an appropriate level of expertise to confirm that it is of an acceptable scientific standard.

# Reviewer report: Reviewer response for version 1

**DOI:** 10.5256/f1000research.195151.r455449

**Published:** 2026-02-18

**Authors:** Mahesh Shenoy

**Affiliations:** 1 Riyadh Elm University, Riyadh, Riyadh Province, Saudi Arabia

Abstract

 Line 2: Change "Oral Potentially Malignant Disorders" to "oral potentially malignant disorders" (lowercase for consistency)

 Line 4: Add "histopathologically" before "confirmed cases"

 Line 7: Change "Chi-square" to "chi-square" (lowercase)

 Line 10: Change "c-Myc, but their expression" to "c-Myc; however, expression"

 Line 11: Change "In OPMDs, positivity" to "In OPMDs, positivity"

 Line 15: Change "supporing" to "supporting"

 Introduction

 Line 3: Change "treatment, identification and prevention" to "treatment, identification, and prevention"

 Line 4: Change "threat worldwide.2 The multifactorial" to "threat worldwide.² The multifactorial"

 Line 6: Change "u-v radiation" to "UV radiation"

 Line 9: Change "Hanahan added polymorphic microbiomes" to "Hanahan identified polymorphic microbiomes"

 Line 10: Remove incomplete sentence fragment "The components of oral microbiota including bacteria, fungi, and viruses, as emerging hallmarks and enabling characteristics."

 Line 14: Change "anti-apoptotic activity" to "anti-apoptotic pathways"

 Line 18: Change "matrixmatalloproteases-9" to "matrix metalloproteinase-9"

 Line 20: Change "through endothelial cadherin(E-cadherin)" to "through E-cadherin"

 Line 22: Change "chromosomes 8q21and and comprised" to "chromosome 8q21 and comprise"

 Materials and Methods

 Line 2: Change "institutional board" to "Institutional Ethics Committee"

 Line 3: Add space after "Ref. No:"

 Line 4: Change "A total 32 samples" to "A total of 32 samples"

 Line 10: Change "phosphate buffer saline" to "phosphate-buffered saline"

 Line 11: Change "Histopathologically confirmed" to "Histopathologically confirmed"

 Line 13: Change "exclusion criteria cases" to "Exclusion criteria included cases"

 Line 20: Change "Immunoscoring for positive cells for c-Myc was done score 1- <25% cells, score 2-25-50% cells, score 3-50-75% cells, score 4- >75% cells.11 For qualitative analysis tissue section was observed" to "Immunoscoring for c-Myc-positive cells was performed as follows: score 1, <25% cells; score 2, 25-50% cells; score 3, 50-75% cells; score 4, >75% cells.¹¹ For qualitative analysis, tissue sections were observed"

 Results

 Line 2: Change "Group I" and "Group II" to "Group I" and "Group II" (maintain consistency)

 Line 3: Add comma after "sex"

 Line 4: Change "Table 1" to "Table 1" (ensure proper formatting)

 Line 6: Change "p = 0.00" to "p < 0.001"

 Line 8: Change "Table 2" to "Table 2"

 Line 9: Change "semi-quantitative" to "semiquantitative"

 Line 13: Remove incomplete table formatting at end of section

 Discussion

 Line 1: Change "species that collectively" to "species that collectively"

 Line 4: Add comma after "2019"

 Line 6: Change "Hanahan, 20221 Hallmarks of Cancer: New Dimensions -Polymorphic Microbiome" to "Hanahan (2022)¹ proposed the polymorphic microbiome as"

 Line 7: Change "This hallmark of cancer influences its other hallmarks" to "This hallmark influences other cancer hallmarks"

 Line 15: Change "Singh AK et al. (2021)16" to "Singh et al. (2021)¹⁶"

 Line 17: Change "with (50.0%) cases" to "with 50.0% of cases"

 Line 22: Change "Sakai et al. (1990),19" to "Sakai et al. (1990)¹⁹"

 Line 25: Change "Graph 2" to "Figure 2"

 Line 30: Change "Pérez-Sayáns et al. (2011)21 and Segura et al. (2013)20" to "Pérez-Sayáns et al. (2011)²¹ and Segura et al. (2013)²⁰"

 Line 35: Add period after "et al" throughout (e.g., "Baral et al." not "Baral et al")

 Line 39: Change "Dang et al. (1999)27" to "Dang et al. (1999)²⁷"

 Line 43: Change "Nagy et al in 1998, Al-Hebshi et al in 2017, Zhao et al in 2017" to "Nagy et al. (1998),²⁸ Al-Hebshi et al. (2017),²⁹ and Zhao et al. (2017)³⁰"

 Line 45: Change "Zhang L et al in 2020" to "Zhang et al. (2020)³¹"

 Line 46: Change "Graph 1" to "Figure 1"

 Line 52: Change "Wu et al. (2016)35" to "Wu et al. (2016)³⁵"

 Line 59: Change "Zhang et al. (2018)36" to "Zhang et al. (2018)³⁶"

 Line 60: Change "1) stimulation" to "(1) stimulation"

 Line 63: Change "Pietrobon G et al. (2021)37" to "Pietrobon et al. (2021)³⁷"

 Conclusion

 Line 1: Change "Our results support that Fusobacterium nucleatum acts" to "Our results support that F. nucleatum acts"

 Line 2: Remove redundant final paragraph (lines 4-7 duplicate the abstract)

 Ethics Approval

 Line 1: Change "Internal Institutional Ethical Committee I.T.S Centre" to "Institutional Ethics Committee of I.T.S Centre"

 Data Availability Statement

 Line 4: Change "provided by" to "properly credited to"

 References

 Ensure all references follow consistent formatting (journal abbreviations, punctuation)

 Reference 11: Verify journal name format

 References 38-39: Update access dates if needed

 General Formatting

 Standardize all superscript reference numbers to use consistent formatting

 Ensure all figure citations in text match figure numbers

 Remove any remaining placeholder or incomplete table text

 Standardize hyphenation of "c-Myc" throughout (currently varies between "c-Myc" and "c-myc")

 Ensure all bacterial species names are italicized throughout (Fusobacterium nucleatum, F. nucleatum)

Is the work clearly and accurately presented and does it cite the current literature?

Yes

If applicable, is the statistical analysis and its interpretation appropriate?

Partly

Are all the source data underlying the results available to ensure full reproducibility?

Yes

Is the study design appropriate and is the work technically sound?

Yes

Are the conclusions drawn adequately supported by the results?

Yes

Are sufficient details of methods and analysis provided to allow replication by others?

Yes

Reviewer Expertise:

Oral pathology

I confirm that I have read this submission and believe that I have an appropriate level of expertise to confirm that it is of an acceptable scientific standard, however I have significant reservations, as outlined above.
